# Early full or near-full enteral feeding in clinically stable preterm infants across healthcare settings: a systematic review and meta-analysis of randomized controlled trials

**DOI:** 10.3389/fmed.2026.1921726

**Published:** 2026-08-17

**Authors:** Li Chen, Yan Gao, Rui Liu, Zuofang Wang, Anzhen Wang, Jian Li, Xiaoyan Jiang

**Affiliations:** 1 The Second Rehabilitation Hospital of Shanghai, Shanghai, China; 2 Shanghai University of Medicine and Health Sciences, Shanghai, China; 3 College of Health, Medicine and Wellbeing, The University of Newcastle, Newcastle, NSW, Australia; 4 West China Second University Hospital, Sichuan University, Chengdu, Sichuan, China

**Keywords:** early full enteral feeding, healthcare settings, meta-analysis, preterm infants, standard care

## Abstract

**Background:**

Preterm infants are at high risk of feeding intolerance. Early full enteral feeding (EFEF) may promote gastrointestinal maturation and reduce hospital stay, but concerns exist about its generalizability across different healthcare contexts.

**Methods:**

We systematically searched multiple databases and included nine randomized controlled trials comparing early full enteral feeding (EFEF), defined as initiation of full enteral feeding immediately after birth without parenteral nutrition or fluid supplementation, with gradual feeding strategies in preterm infants. Subgroup analyses were performed according to healthcare settings (high-income vs. low- and middle-income countries). The primary outcome was length of hospital stay.

**Results:**

Nine randomized controlled trials involving 2,777 infants were included. EFEF reduced length of hospital stay (MD = −4.41 days, 95% CI: −7.05 to −1.76), time to regain birth weight (MD = −2.29 days, 95% CI: −3.66 to −0.91), and time to achieve full enteral feeding (MD = −1.58 days, 95% CI: −2.32 to −0.84). No statistically significant difference was observed in feeding intolerance (RR = 0.78, 95% CI: 0.49–1.22), necrotizing enterocolitis (RR = 0.89, 95% CI: 0.38–2.09), invasive infection (RR = 0.82, 95% CI: 0.43–1.57), mortality (RR = 1.08, 95% CI: 0.55–2.09), or hypoglycaemia (RR = 1.53, 95% CI: 0.42–5.59), although the confidence intervals for serious adverse outcomes were wide. Subgroup analyses showed no statistically significant interaction between healthcare settings for either length of hospital stay (*P* for interaction = 0.79) or mortality (*P* for interaction = 0.90).

**Conclusions:**

EFEF may shorten hospital stay and accelerate feeding-related milestones in clinically stable preterm infants. However, the certainty of evidence was low to very low, and estimates for serious adverse outcomes were imprecise. The current evidence therefore does not establish the safety of EFEF or determine whether its effects differ across healthcare settings.

**Systematic review registration:** https://www.crd.york.ac.uk/PROSPERO/, identifier CRD420251251351.

## Introduction

Preterm infants face a high risk of feeding intolerance due to extreme immaturity of the gastrointestinal system and deficiencies in immune defense mechanisms (1, 2). Consequently, identifying the optimal enteral feeding strategy for preterm infants remains a central challenge in neonatal intensive care (3–5). Achieving full enteral nutrition as early as possible in life, referred to as early full enteral feeding (EFEF), may promote gastrointestinal functional maturation through hormonal stimulation and reduce the risk of iatrogenic infection by decreasing the use of central venous catheters (4, 6). However, in clinical practice, driven by concerns regarding necrotizing enterocolitis (NEC) and feeding intolerance, “gradual feeding” remains the predominant strategy, particularly in the management of extremely preterm infants (7, 8). This tension between the anticipated physiological benefits and concerns about pathological risks has resulted in substantial global variation in feeding practices (4, 18).

Over the past decade, multiple randomized controlled trials (RCTs) (9–14), primarily conducted in low- and middle-income countries (LMICs), have reported that initiating near-full or full-dose enteral feeding from the first day of life not only accelerates gastrointestinal maturation, shortens the time to achieve full enteral feeding, and promotes weight recovery, but also significantly reduces length of hospital stay, without increasing the risks of NEC, mortality, or other feeding-related complications. A previous review included six randomized controlled trials (9–14) involving 526 infants, all conducted in neonatal care facilities in India, and concluded that the available evidence was insufficient to determine with certainty the effects of early full enteral feeding on important clinical outcomes, including growth and necrotizing enterocolitis (NEC). The certainty of evidence was rated as low or very low because of methodological limitations, inconsistency, and imprecision. Nevertheless, the findings suggested that early full enteral feeding may be feasible in clinically stable preterm infants and highlighted the need for larger, higher-quality trials to clarify its benefits and risks (18).

Since the publication of that review, several large randomized controlled trials have been conducted in diverse healthcare settings, including high-income countries (HICs). Notably, the multicenter FEED1 trial (15) conducted in the United Kingdom enrolled more than 2,000 preterm infants and substantially expanded the evidence base. While this study demonstrated that early full enteral feeding reduced exposure to parenteral nutrition, it did not shorten length of hospital stay or improve growth outcomes. Other contemporary studies have reported varying findings. For example, Alshaikh et al. (16) reported a reduction in hospital stay, whereas Razzaghy et al. (17) demonstrated favorable cost-effectiveness without a significant reduction in length of stay. Collectively, these newer studies suggest that although the safety profile of early full enteral feeding appears broadly consistent across settings, its effectiveness may be influenced by differences in healthcare systems, discharge practices, and clinical management strategies.

Overall, the certainty of the available evidence regarding early full enteral feeding remains limited, and potential differences across healthcare contexts have not been adequately examined, particularly across different healthcare contexts. Previous meta-analyses were based predominantly on studies from low- and middle-income countries (LMICs), did not include recently published large-scale trials, and did not formally examine whether healthcare system characteristics modify the effects of early full enteral feeding. Consequently, important questions remain unresolved: Does early full enteral feeding provide consistent clinical benefits across different healthcare systems? Are the currently available data sufficient to support its safety and broader implementation in neonatal practice?

Therefore, we conducted an updated systematic review and meta-analysis of randomized controlled trials to evaluate the efficacy and safety of early full or near-full enteral feeding compared with conventional gradual feeding strategies in clinically stable preterm infants. Particular attention was given to healthcare setting as a potential source of heterogeneity, with subgroup analyses comparing outcomes between HICs and LMICs.

## Methods

This study was conducted in strict adherence to the Preferred Reporting Items for Systematic Reviews and Meta-Analyses (PRISMA) guidelines (20) and has been registered in the international systematic review registry PROSPERO (https://www.crd.york.ac.uk/PROSPERO) with registration number CRD420251251351.

### Information sources and search strategy

We systematically searched five electronic databases, including the Cochrane Central Register of Controlled Trials (CENTRAL), PubMed, MEDLINE, Web of Science, and Embase. ClinicalTrials.gov was searched separately as a clinical trial registry. The search period spanned from the inception of each database to October 17, 2025, and included preprints and articles published online ahead of print. In addition to the database search, we screened the reference lists of included studies and relevant systematic reviews and searched conference abstracts, dissertations, and other gray-literature sources. The search was conducted in English, with no restriction on country or region. Two reviewers (LC and YG) independently and blindly performed the literature screening, with any disagreements resolved through discussion. In cases where consensus could not be reached, a third reviewer (XJ) was consulted for a final decision. Studies were considered eligible if the title, abstract, full text, substance names, subject terms, keywords, or unique identifiers included the following Medical Subject Headings (MeSH) terms and keywords: “Early Full Enteral Feeding,” “Gradual Feeding,” “Preterm Infants,” “Premature infant,” “Premature baby,” and “Low birth weight infant.”

### Inclusion criteria

Based on the PICOS framework, the inclusion criteria were as follows:

Population (P): preterm infants, defined as those born < 37 weeks of gestational age or with a birth weight < 2500 g.

Intervention (I): early full enteral feeding (EFEF), defined as initiation of near-full or full enteral feeding within the first day of life, with minimal or no parenteral nutrition supplementation.

Comparison (C): conventional or gradual feeding strategies, involving lower initial enteral feeding volumes with progressive advancement and supplemental parenteral nutrition or intravenous fluids as clinically indicated.

Outcomes (O): the primary outcome was length of hospital stay. Secondary outcomes included time to regain birth weight, time to achieve full enteral feeding, incidence of necrotizing enterocolitis (NEC), feeding intolerance, invasive infection, mortality, and hypoglycaemia.

Study Type (S): only randomized controlled trials (RCTs) were included.

### Study selection process

The initial screening of titles and abstracts was independently conducted by two authors (LC and YG). After the initial screening, full-text articles of potentially eligible studies were reviewed. Disagreements during the screening process were resolved through discussion to minimize bias. In case of unresolved discrepancies, a third reviewer (XJ) made the final decision.

### Data extraction

Two reviewers (LC and YG) independently extracted data using a pre-designed data extraction form. Data extracted included: authors, publication year, country, study design, sample size, length of hospital stay, time to regain birth weight, incidence of necrotizing enterocolitis (NEC), incidence of feeding intolerance, infection risk, mortality, and hypoglycemia. Long-term outcomes were also included if reported. All extracted data were cross-checked by a third reviewer (XJ).

### Risk of bias assessment

Two reviewers (LC and YG) independently assessed the risk of bias using the Cochrane Risk of Bias 2 (ROB 2) (21) tool. This tool evaluates methodological quality across five domains: randomization process, deviation from intended interventions, missing outcome data, outcome measurement, and selection of reported results. Studies were classified as high risk if one or more domains were rated as “high risk,” as low risk if all domains were rated as “low risk,” and as unclear risk if any domain was rated as “some concerns.” Any disagreements were resolved through discussion, with a third reviewer providing final judgment if necessary.

### Quality of evidence assessment

The certainty of evidence for primary and key secondary outcomes (including length of hospital stay, necrotizing enterocolitis, mortality, invasive infections, and time to regain birth weight) was assessed using the GRADE approach (22). Two authors (LC and YG) independently evaluated the quality of evidence across the dimensions of study design, risk of bias, inconsistency, imprecision, and indirectness, and classified the evidence as high, moderate, low, or very low. Disagreements were first resolved through discussion, and if consensus could not be reached, a third reviewer made the final decision.

### Data analysis

All statistical analyses were conducted using the pre-specified meta-analysis plan, with Review Manager software (RevMan 5.4.1, Cochrane Collaboration). Due to clinical and methodological differences across studies, all pooled effect estimates were calculated using a random-effects model to better reflect heterogeneity and provide more conservative, generalizable estimates (19).

For binary outcomes, relative risks (RR) and 95% confidence intervals (CIs) were calculated; for continuous outcomes, mean differences (MD) were used. A *P*-value of < 0.05 (two-sided test) was considered statistically significant. Heterogeneity was quantified using the I^2^ statistic (23), with thresholds defined as follows: *I*^2^ < 25% as low, 25%−50% as moderate, 50%−75% as substantial, and >75% as high heterogeneity. As fewer than 10 studies were included, formal assessment of publication bias was not performed. Funnel plots were inspected only as an exploratory visual tool (24, 25).

### Sensitivity analysis

To test the robustness of the results, we performed the following sensitivity analyses:

(1) Changing to a fixed-effect model;
(2) Excluding all gray-literature studies;
(3) Sequentially excluding each study to observe whether the pooled results changed significantly.

### Subgroup analysis

To further explore whether the effects of early full enteral feeding on length of hospital stay and mortality varied across different healthcare settings, we conducted subgroup analyses for both outcomes, stratified by country income level into high-income countries (HICs) and low- and middle-income countries (LMICs).

## Results

**Figure 1:**
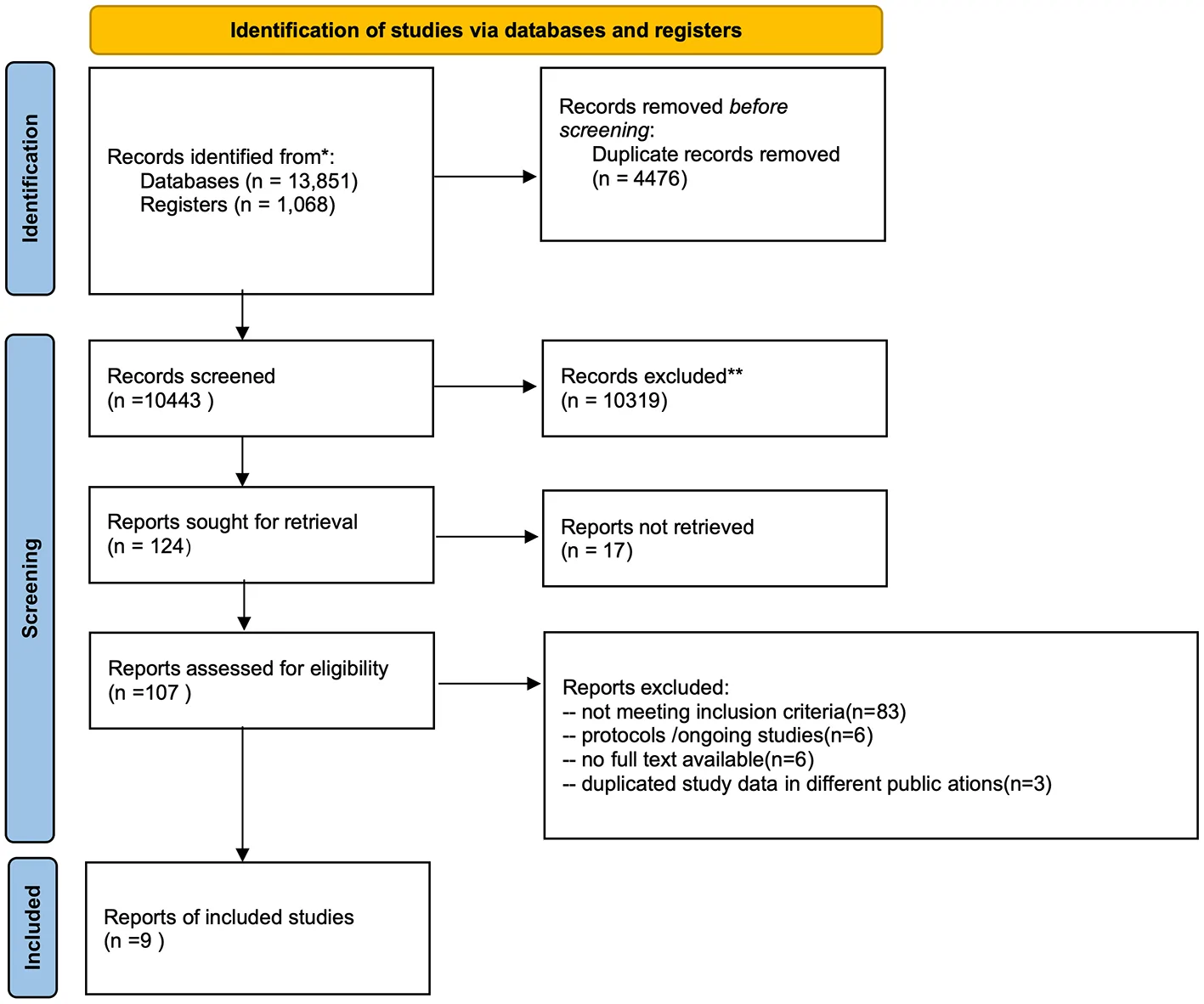
Flow diagram of the included studies.

Our initial literature search identified 14,919 records. After removing duplicates, 10,443 independent articles remained for screening. After strict evaluation of titles, abstracts, and full texts, a total of 9 randomized controlled trials (RCTs) (9–17) were included in this systematic review (Figure 1). Table 1 summarizes the basic characteristics of the trials included in this analysis.

**Table 1:** Characteristics of the included RCTs.

References	Country	Gestational age at birth (weeks), mean ± SD		Multiple births and SGA infants, n/N (%)		Birth weight (g), mean ± SD		Male (%)		Sample size (Intervention/Control)		Parenteral nutrition use allowed?	Milk type		Feeding type	
		EFEF	Conventional	Multiple births	SGA infants	EFEF	Conventional	EFEF	Conventional	EFEF	Conventional		EFEF	Conventional	EFEF	Conventional
Sanghvi et al. (14)	India	31.8 ± 2.4	31.2 ± 1.8	NA	9/46 (19.6%)	1350 ± 110	1320 ± 110	52.1	56.5	23	23	No, in EEEF; Yes (IV fluids only) in control	formula or fortified breast milk	MOM + Preterm formula + HMF; No DHM	80 mL/kg/d D1	30 mL/kg/d + IV fluids
Chetry et al. (11)	India	NA	NA	NA	NA	NA	NA	NA	NA	31	33	NA	formula or fortified breast milk	formula or fortified breast milk	80 mL/kg/d D1	20 mL/kg minimal feeds + IV fluids
Bora et al. (10)	India	35.45 ± 0.2572	34.808 ± 1.91	NA	64/103 (62.1%)	1378 ± 17	1337 ± 20	0.237	0.383	51	52	No, in EEEF; Yes, IV fluids only in control	MOM or DHM+cow milk based HMF	MOM or DHM+cow milk based HMF	80 mL/kg/day from birth, no PN/IV fluids allowed	20 mL/kg/day + IV fluids (10% dextrose) until ≥100 mL/kg/day
Nangia et al. (13)	India	31 (28–34)	32 (28–34)	NA	54/180 (30.0%)	1323 ± 125	1305 ± 132	49.4	47.2	91	89	No, neonate received parenteral nutrition	MOM or formula	MOM or formula	Early full enteral feeding on day 1 (80 mL/kg)	20 mL/kg minimal feeds + IV fluids
Shanmugam et al., (9)	India	NA	NA	NA	11/69 (16.0%)	1000–1500	1000–1500	NA	NA	34	35	No, in EEEF; Yes (IV fluids only) in control	MOM or DHM	MOM or DHM	60 mL/kg/day → +20 mL/kg/day	Progressive feeds with IV fluids
Jajoo et al. (12)	India	31.8 ± 1.3	31.6 ± 1.4	NA	21/60 (35.0%)	1353.8 ± 138.2	1333.8 ± 158.0	54.8	51.7	31	29	Intervention: No; Control: Yes, until 100 mL/kg/d	MOM 64.5%, formula 0%, mixed 35.5%	MOM 62.1%, formula 6.9%, mixed 31.0%	Early total enteral feeding, 80 mL/kg/day on day 1 → advance 20 mL/kg/day	Gradual advancement, 30 mL/kg/day on day 1 → advance 20 mL/kg/day
Razzaghy et al. (17)	USA	31 (30–32)	30 (29–32)	NA	5/102 (4.9%)	1571 ± 317	1385 ± 336	48	52	48	49	Yes, all infants got IV fluids at birth; TPN 4% vs 12%	MOM or DHM+formula	MOM or DHM+formula	60–80 mL/kg/day day 1	20–30 mL/kg/day gradual
Alshaikh et al. (16)	Canada	31.5 ± 1.1	31.6 ± 0.9	11/70 (15.7%)	6/70 (8.6%)	1732 ± 291	1708 ± 364	0.486	0.571	35	35	Yes, but minimized in EEEF; routine in control	MOM or DHM	MOM or DHM	60–80 mL/kg/day from birth. no PN unless already started	20–30 mL/kg/day + IV fluids/PN until ≥100 mL/kg/day
Ojha et al., (15)	United Kingdom	31.7 ± 0.8	31.7 ± 0.8	656/2088 (31.4%)	242/2088 (11.6%)	1626.0 ± 301.8	1617.1 ± 295.2	52.7	51.9	1047	1041	No, PN allowed unless intolerance or clinical concerns; Routine PN/IV fluids	MOM or DHM+formula	MOM or DHM+formula	60–80 mL/kg/day from birth, no PN unless already started	≤ 30 mL/kg/day+IV fluids or PN

MOM, mother's own milk; DHM, donor human milk; HMF, human milk fortifier, SGA, small for gestational age.

These studies were published between 2013 and 2025, with sample sizes ranging from 46 to 2,088 participants. There was some degree of heterogeneity in subject characteristics and study protocols across the included studies (9–17). Although all participants were preterm infants, there were differences in gender distribution and birth weight. Across the nine included trials (9–17), 2,777 clinically stable preterm infants were enrolled. The reported gestational ages were generally centered around 32 weeks, although the eligibility ranges of most trials crossed the 32-week threshold. Birth weights were predominantly within the low- or very-low-birth-weight ranges. One trial exclusively enrolled infants below 32 weeks, one exclusively enrolled infants at or above 32 weeks, and the remaining seven included infants across both gestational-age categories without reporting separate subgroup data. Additionally, the choice of milk type for early feeding interventions varied between studies. Six trials (11–15, 17) primarily used formula milk as a supplement when maternal breast milk was insufficient, while three other trials (9, 10, 16) preferred donor breast milk as a supplement. For infants with specific high nutritional needs, two studies (9, 10) included breast milk fortifiers as part of the intervention. Detailed gestational-age and birth-weight characteristics, the number of small-for-gestational-age infants where reported, and the principal clinical eligibility and stability criteria are presented in Table 1.

**Figure 2:**
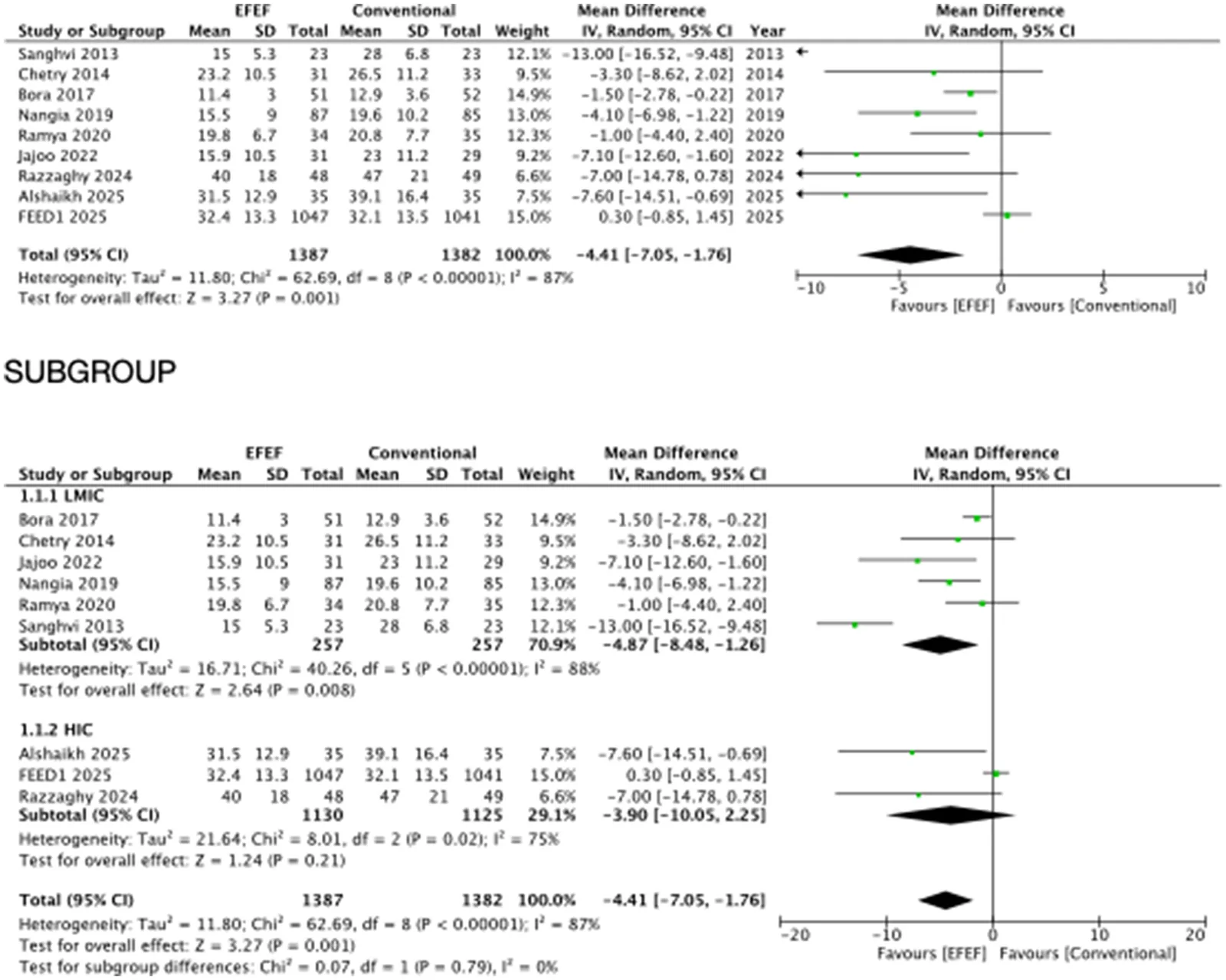
Forest plot of the effect of early enteral feeding (EFEF) vs. conventional feeding on the length of hospital stay in preterm infants (subgroup analysis by economic development level of countries/regions).

Several studies reported the following outcomes. Nine studies (9–17) evaluated length of hospital stay, with a total of 1,387 infants in the EFEF group and 1,382 infants in the conventional feeding group. The pooled result showed that EFEF shortened hospital stay by an average of 4.41 days compared with conventional feeding (MD = −4.41; 95% CI: −7.05 to −1.76; Figure 2).

**Figure 3:**
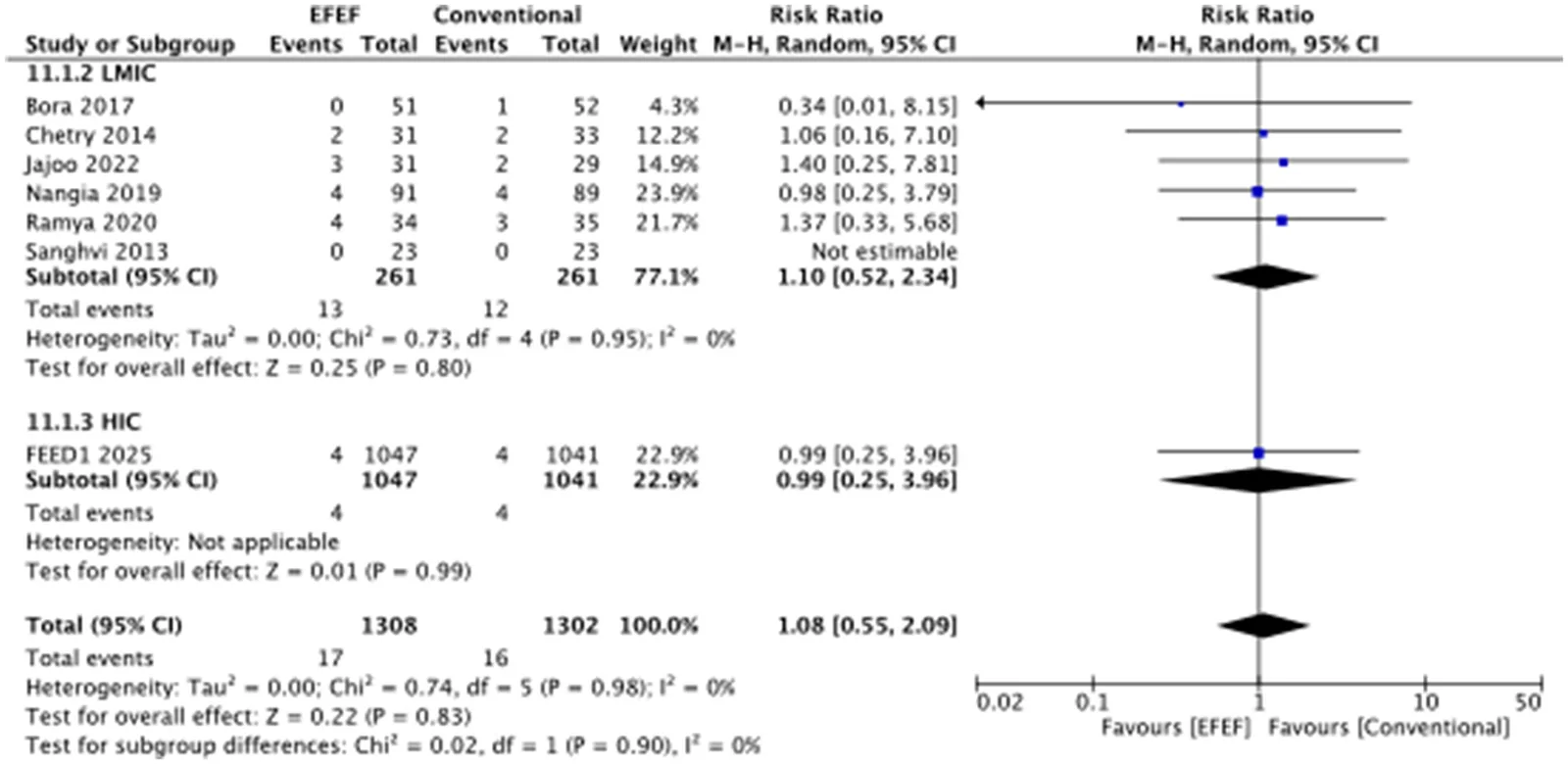
Forest plot of the effect of early enteral feeding (EFEF) vs. conventional feeding on mortality in preterm infants (subgroup analysis by the economic development level of countries/regions).

In addition, infants in the EFEF group regained their birth weight an average of 2.29 days earlier than those in the conventional feeding group (MD = −2.29; 95% CI: −3.66 to −0.91; Figure S1). The time to achieve full enteral feeding was also shortened by an average of 1.58 days in the EFEF group (MD = −1.58; 95% CI: −2.32 to −0.84; Figure S2). No statistically significant difference was observed in feeding intolerance between the groups (RR = 0.78; 95% CI: 0.49–1.22; Figure S3). Similarly, the incidence of necrotizing enterocolitis (NEC) did not differ significantly between the groups. NEC occurred in 0.93% of infants in the EFEF group (13/1,391) and 1.08% of infants in the conventional feeding group (15/1,386; RR = 0.89; 95% CI: 0.38–2.09; Figure S4). No statistically significant differences were observed in invasive infection (RR = 0.82; 95% CI: 0.43–1.57; Figure S5), mortality (RR = 1.08; 95% CI: 0.55–2.09; Figure 3), or hypoglycaemia (RR = 1.53; 95% CI: 0.42–5.59; Figure S6) between the groups. However, the confidence intervals for these uncommon adverse outcomes were wide, indicating that the safety estimates remained imprecise.

We performed a pre-specified subgroup analysis to explore the potential influence of healthcare system context on length of hospital stay. The interaction test showed no statistically significant subgroup interaction between low- and middle-income countries (LMICs) and high-income countries (HICs; *P* for interaction = 0.79). In the LMIC subgroup, EFEF was associated with a statistically significant reduction in hospital stay (MD = −4.87; 95% CI: −8.48 to −1.26; *P* = 0.008). In the HIC subgroup, a similar direction of effect was observed (MD = −3.90), although the result did not reach statistical significance (95% CI: −10.05 to 2.25; *P* = 0.21; Figure 2). The wider confidence interval in the HIC subgroup may reflect greater variability across studies and limited statistical power. Therefore, the non-significant interaction should not be interpreted as confirming equivalent effects across healthcare settings.

We additionally performed a subgroup analysis of mortality according to healthcare setting. In the LMIC subgroup, no statistically significant difference in mortality was observed between EFEF and conventional feeding (RR = 1.10; 95% CI: 0.52–2.34). Similarly, no statistically significant difference was observed in the HIC subgroup (RR = 0.99; 95% CI: 0.25–3.96). The overall pooled estimate also showed no statistically significant difference in mortality (RR = 1.08; 95% CI: 0.55–2.09), and the test for subgroup differences was not statistically significant (*P*-interaction = 0.90; Figure 3). However, these findings should be interpreted cautiously because mortality events were rare, the confidence intervals were wide, and only one study contributed to the HIC subgroup.

Sensitivity analyses using a fixed-effect model, excluding the three gray-literature studies, and sequentially omitting each individual study yielded conclusions consistent with the primary analysis (Table S1). The funnel plot showed slight asymmetry. However, because fewer than 10 studies were available and substantial heterogeneity was present, this observation was considered exploratory and was not interpreted as reliable evidence of publication bias (Figure S7).

The risk-of-bias assessment results are summarized in Figure S8 of the supplementary material. All nine included trials were classified as being at high risk of bias, primarily due to insufficient outcome measurement and selective reporting. The certainty of evidence for each endpoint is presented in Table 2. The certainty was rated as low for necrotizing enterocolitis, feeding intolerance, hypoglycaemia, and mortality, and as very low for length of hospital stay, time to regain birth weight, invasive infection, and time to achieve full enteral feeding. The evidence was downgraded primarily because of risk of bias, inconsistency, and serious imprecision.

**Table 2:** Summary of findings and strength of evidence.

Outcome	No. of patients (Trials)	Relative effect, risk ratio (95% CI)	Absolute effect estimates (per 1,000)			Quality of the evidence
			EFEF	Conventional	Difference	
The primary outcome						
Length of hospital stay	2,769 (9)	−4.41 (−7.05, −1.76)				Very low^*†^
The secondary outcome						
Days to regain birth weight	575 (7)	−12.29 (−13.66, −10.91)				Very low^*†^
Necrotizing enterocolitis	2,777 (9)	0.89 (0.38, 2.09)	9	11	−1 [−7, 12]	Low^†^
Feed intolerance	518 (6)	0.78 (0.49, 1.22)	162	212	−50 [−108, 47]	Low^*†^
Invasive infection	2,572 (7)	0.82 (0.43, 1.57)	34	36	−6 [−20, 20]	Very low^*†^
Hypoglycaemia	288 (3)	1.53 (0.42, 5.59)	34	21	11 [−12, 96]	Low^†^
Mortality	2,610 (7)	1.08 (0.55, 2.09)	13	12	1 [−6, 13]	Low^†^
Time to full enteral feeding	2,530 (5)	−1.58 (−2.32, −0.84)				Very low^*†^

CI, confidence interval; RR, risk ratio.

^*^ Inconsistency; ^†^Serious imprecision (wide confidence intervals crossing no effect).

## Discussion

In this updated meta-analysis, early full enteral feeding was associated with shorter hospital stay and faster achievement of feeding-related milestones. No statistically significant increase in major adverse outcomes was detected. However, all included trials were judged to be at high risk of bias, the certainty of evidence was low to very low, and estimates for uncommon serious outcomes were imprecise. Therefore, the findings should not be interpreted as definitive evidence of safety. Although the point estimates for length of hospital stay were in the same direction in HIC and LMIC subgroups, the evidence was insufficient to establish consistency across healthcare settings.

Compared with the previous review, the present meta-analysis substantially expands the evidence base by including nine randomized controlled trials (9–17) involving 2,777 infants, including three recent studies conducted in high-income countries and the large multicentre FEED1 trial (15). In addition to increasing the sample size and geographical diversity of the available evidence, this review formally examined healthcare setting as a potential source of heterogeneity and incorporated newer data on feeding progression, length of hospital stay, and major adverse outcomes. However, these advances do not eliminate the important limitations of the current evidence. The certainty of evidence remains low to very low, all included trials were judged to be at high risk of bias, and estimates for uncommon outcomes such as necrotizing enterocolitis and mortality remain imprecise.

These methodological limitations directly reduced the precision of the effect estimates and weakened the reliability of the previous conclusions. In addition, several recent randomized controlled trials reported inconsistent findings, further highlighting the uncertainty in this area. Nevertheless, by incorporating these recent and relatively large-scale trials (15–17), our analysis increased the available sample size and improved the precision of the pooled estimates. The results suggest that early full enteral feeding may shorten the length of hospital stay and reduce the time required to regain birth weight in preterm infants. These findings expand the existing evidence base and indicate that the potential benefits of early full enteral feeding are becoming clearer as additional data accumulate. We also conducted subgroup analyses according to country income level. A reduction in the length of hospital stay was observed in the LMIC subgroup, although this finding should be interpreted cautiously because of the limited number of studies and the potential for insufficient statistical power in subgroup comparisons. The observed improvements in feeding-related and recovery outcomes may be supported by several biologically plausible mechanisms. Early exposure to enteral nutrients stimulates intestinal epithelial proliferation and mucosal barrier maturation, reduces intestinal atrophy and microbial dysbiosis, and provides amino acids and immunologically active components in human milk that may suppress pro-inflammatory responses, thereby limiting organ injury and infection-related complications (4, 6). Adequate enteral nutrition may also promote metabolic stability, facilitate weight gain, and enable earlier achievement of discharge-related milestones, without clear evidence of an increased risk of necrotizing enterocolitis or hypoglycaemia. Furthermore, early full enteral feeding may reduce exposure to parenteral nutrition and invasive catheters, shorten the duration of intensive care, and minimize the metabolic disturbances, delayed gastrointestinal adaptation, and procedure-related inflammatory stress associated with parenteral nutrition and invasive interventions (15, 26), all of which may otherwise delay discharge and the achievement of full enteral feeding.

Although the pooled estimate reached statistical significance in the LMIC subgroup but not in the HIC subgroup, a difference in statistical significance between subgroups does not itself indicate a difference in treatment effect. The test for interaction was not statistically significant (*P* = 0.79). However, because only three HIC trials were available and the confidence interval waswide, the evidence remains insufficient to determine whether country income category modifiesthe effect of EFEF on hospital stay.

In some LMIC settings, feeding practices and nutritional milestones play an important role in the clinical management of very low birthweight and very preterm infants (27). Since EFEF accelerates the achievement of both, the intervention effect is more directly translated into a shorter hospital stay, leading to a clear benefit signal statistically. In contrast, in HICs, discharge timing is influenced by multiple factors, which often constitute competing “rate-limiting steps” (28). Even though EFEF helps infants achieve full enteral feeding earlier, discharge may still be delayed due to non-nutritional factors such as respiratory stability, neurodevelopmental monitoring, or family care preparation (29). These factors introduce more variability into the outcome of “length of stay,” thus attenuating the nutritional benefit signal of EFEF and widening the confidence interval (29). Therefore, in resource-rich settings, the value of EFEF may be better reflected in “time to achieve full enteral feeding” or “reduced use of parenteral nutrition” rather than merely in reduced length of stay.

The limitations of the existing evidence include a small number of non-blinded trials, low to very low certainty of evidence, substantial imprecision and heterogeneity, and the inclusion of three non-peer-reviewed gray-literature studies. Additionally, the included studies were predominantly conducted in relatively stable low-birth-weight preterm infants from middle-income countries, limiting the generalizability of the findings. Extremely preterm infants, particularly those born at < 30 weeks of gestation, and extremely low birth weight infants (< 1,000 g) were underrepresented in the included trials. Small-for-gestational-age infants and multiple births were also incompletely reported, and subgroup-specific outcome data were unavailable. These populations may differ substantially in gastrointestinal maturity, feeding tolerance, baseline risks of necrotizing enterocolitis and other adverse outcomes, and dependence on parenteral nutritional support. Therefore, the present findings are most applicable to clinically stable preterm infants and should not be extrapolated without caution to more immature or clinically complex populations. Gestational age is an important determinant of feeding tolerance and clinical outcomes in preterm infants (30). However, most included trials enrolled infants across different gestational-age categories and did not report outcomes separately by gestational age. The lack of gestational-age-stratified outcome data therefore represents an important limitation of the current evidence and a priority for future research. Furthermore, reporting of multiple births, small-for-gestational-age status, and other clinically important neonatal characteristics was incomplete, and outcomes were not reported separately for these subgroups. Therefore, potential differences in the effects of EFEF according to these characteristics could not be assessed. Although recent large-scale trials have substantially expanded the evidence base, certainty regarding safety outcomes remains limited because serious adverse events, such as necrotizing enterocolitis and mortality, were rare and the corresponding estimates remained imprecise. Therefore, while the available evidence did not demonstrate a statistically significant increase in risk associated with early full enteral feeding, further adequately powered trials are required before definitive conclusions regarding safety can be drawn. Moreover, further clarification is needed regarding the effects of different supplemental milk types, particularly donor human milk vs. formula (31, 32). Future studies should compare different milk supplementation strategies, report outcomes according to standardized gestational-age categories, and include more infants with birth weights < 1,000 g or gestational ages < 30 weeks to improve the applicability of the evidence and evaluate long-term outcomes (17, 26).

## Conclusion

EFEF may shorten hospital stay and accelerate feeding-related milestones in clinically stable preterm infants. However, given the low to very low certainty of evidence and imprecise estimates for serious adverse outcomes, its safety remains uncertain. Although the direction of effect estimates for hospital stay was similar between HIC and LMIC settings, the limited number of available trials, particularly in HICs, prevents determination of whether healthcare setting modifies the effect of EFEF. Further high-quality trials across diverse healthcare environments are required before EFEF can be considered for broader routine implementation.
