# Supplementary material for: Early full or near-full enteral feeding in clinically stable preterm infants across healthcare settings: a systematic review and meta-analysis of randomized controlled trials
Source: Front Med (Lausanne). 2026 Aug 17;13:1921726. doi: 10.3389/fmed.2026.1921726 (PMC13526999; doi:10.3389/fmed.2026.1921726)
Supplement: Supplementary file 1 [file Data_Sheet_1.docx]

**Appendix**

Figure S1. Forest plot of the effect of early enteral feeding (EFEF) versus conventional feeding on the days to regain birth weight in preterm infants

Figure S2. Forest plot of the effect of early enteral feeding (EFEF) versus conventional feeding on the time to full enteral feeding in preterm infants

Figure S3. Forest plot of the effect of early enteral feeding (EFEF) versus conventional feeding on the incidence of feed intolerance in preterm infants

Figure S4. Forest plot of the effect of early enteral feeding (EFEF) versus conventional feeding on the incidence of necrotizing enterocolitis (NEC) in preterm infants

Figure S5. Forest plot of the effect of early enteral feeding (EFEF) versus conventional feeding on the incidence of invasive infection in preterm infants

Figure S6. Forest plot of the effect of early enteral feeding (EFEF) versus conventional feeding on the incidence of hypoglycaemia in preterm infants

Figure S7: Funnel Plot Assessing Heterogeneity in the Meta-Analysis of Hospital Length of Stay

Figure S8: Risk of bias summary

Table S1: Sensitivity analysis of meta-analysis

Figure S1.Forest plot of the effect of early enteral feeding (EFEF) versus conventional feeding on the days to regain birth weight in preterm infants

Figure S2.Forest plot of the effect of early enteral feeding (EFEF) versus conventional feeding on the time to full enteral feeding in preterm infants

Figure S3.Forest plot of the effect of early enteral feeding (EFEF) versus conventional feeding on the incidence of feed intolerance in preterm infants

Figure S4.Forest plot of the effect of early enteral feeding (EFEF) versus conventional feeding on the incidence of necrotizing enterocolitis (NEC) in preterm infants

Figure S5.Forest plot of the effect of early enteral feeding (EFEF) versus conventional feeding on the incidence of invasive infection in preterm infants

Figure S6.Forest plot of the effect of early enteral feeding (EFEF) versus conventional feeding on the incidence of hypoglycaemia in preterm infants

Figure S7: Funnel Plot Assessing Heterogeneity in the Meta-Analysis of Hospital Length of Stay

FigureS8: Risk of bias summary Domains:

| Study | Risk of bias domains | | | | | |
| --- | --- | --- | --- | --- | --- | --- |
|  | D1 | D2 | D3 | D4 | D5 | Overall |
| Sanghvi et al. 2013 |  |  |  |  |  |  |
| Chetry et al. 2014 |  |  |  |  |  |  |
| Bora et al. 2017 |  |  |  |  |  |  |
| Nangia et al. 2019 |  |  |  |  |  |  |
| Ramya et al. 2020 |  |  |  |  |  |  |
| Jajoo et al. 2022 |  |  |  |  |  |  |
| Razzaghy et al. 2024 |  |  |  |  |  |  |
| Alshaikh et al. 2025 |  |  |  |  |  |  |
| FEED1 et al. 2025 |  |  |  |  |  |  |

D1: Bias arising from the randomization process.

D2: Bias due to deviations from intended intervention.

D3: Bias due to missing outcome data.

D4: Bias in measurement of the outcome.

D5: Bias in selection of the reported result.

:Low risk of bias

:some concerns risk of bias

:High risk of bias

Table S1: Sensitivity analysis of meta-analysis

|  | **NO. patients(trials)** | **MD** | **95%CI** |
| --- | --- | --- | --- |
| **All trials** | 2769(9) | -4.41 | -7.05, -1.76 |
| **Using fixed-effect models** | 2769(9) | -1.67 | -2.43, -0.92 |
| **Excluding all grey-literature studies** | 2576(6) | -4.88 | -8.27, -1.49 |
| **Excluding each randomized control trial in turn** |  |  |  |
| **Excluding Sanghvi et al.2013** | 2723(8) | -2.56 | -4.36,-0.75 |
| **Excluding Chetry et al.2014** | 2705(8) | -4.54 | -7.38, -1.71 |
| **Excluding Bora et al. 2017** | 2666(8) | -5.09 | -8.82, -1.37 |
| **Excluding Nangia et al. 2019** | 2597(8) | -4.49 | -7.43,-1.55 |
| **Excluding Ramya et al. 2020** | 2700(8) | -4.93 | -7.87,-1.98 |
| **Excluding Jajoo et al.2022** | 2709(8) | -4.13 | -6.88,-1.37 |
| **Excluding Razzaghy et al.2024** | 2672(8) | -4.22 | -6.96,-1.49 |
| **Excluding Alshaikh et al.2025** | 2699(8) | -4.14 | -6.88,-1.41 |
| **Excluding FEED1 et al.2025** | 681(8) | -5.30 | -8.47,-2.12 |
